# Supplementary material for: Central and Effector Memory Human CD4+ and CD8+ T Cells during Cutaneous Leishmaniasis and after In Vitro Stimulation with Leishmania (Viannia) braziliensis Epitopes
Source: Vaccines (Basel). 2023 Jan 11;11(1):158. doi: 10.3390/vaccines11010158 (PMC9861845; doi:10.3390/vaccines11010158)
Supplement: Supplementary file 1 [file vaccines-11-00158-s001.zip › vaccines-2119352-supplementary.pdf]

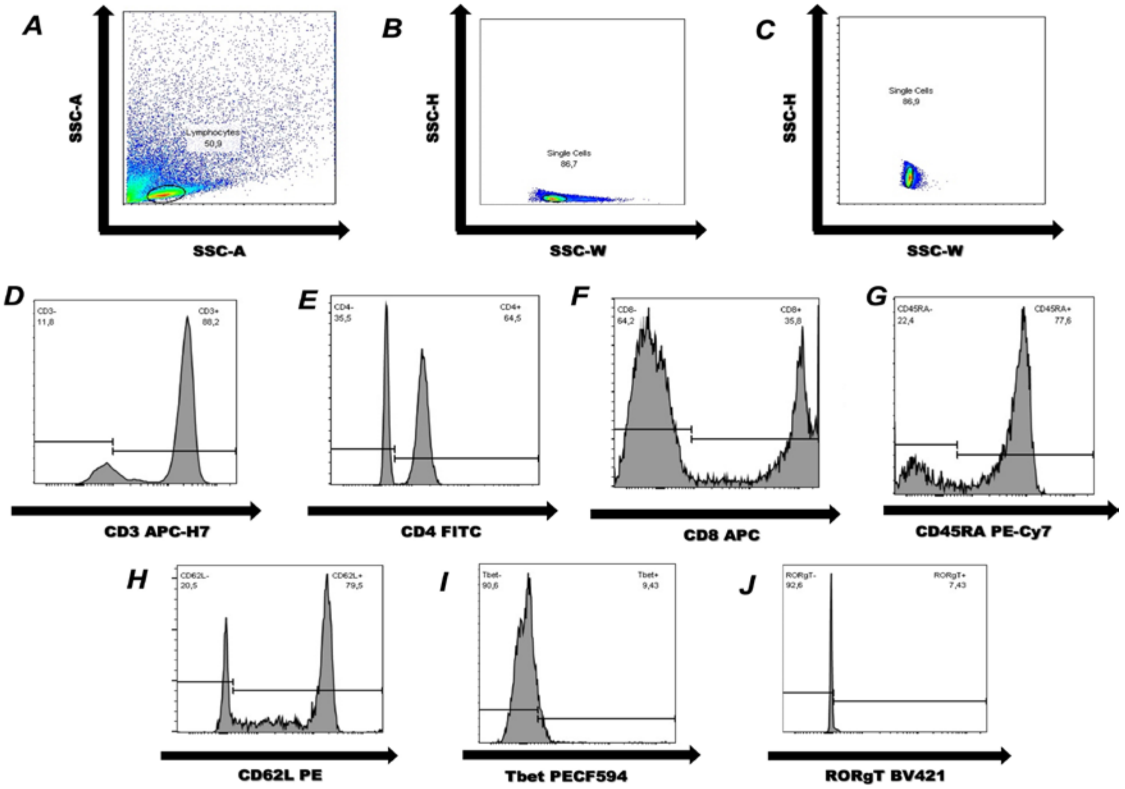


**Figure S1.** Gating strategy for immunological memory panel.

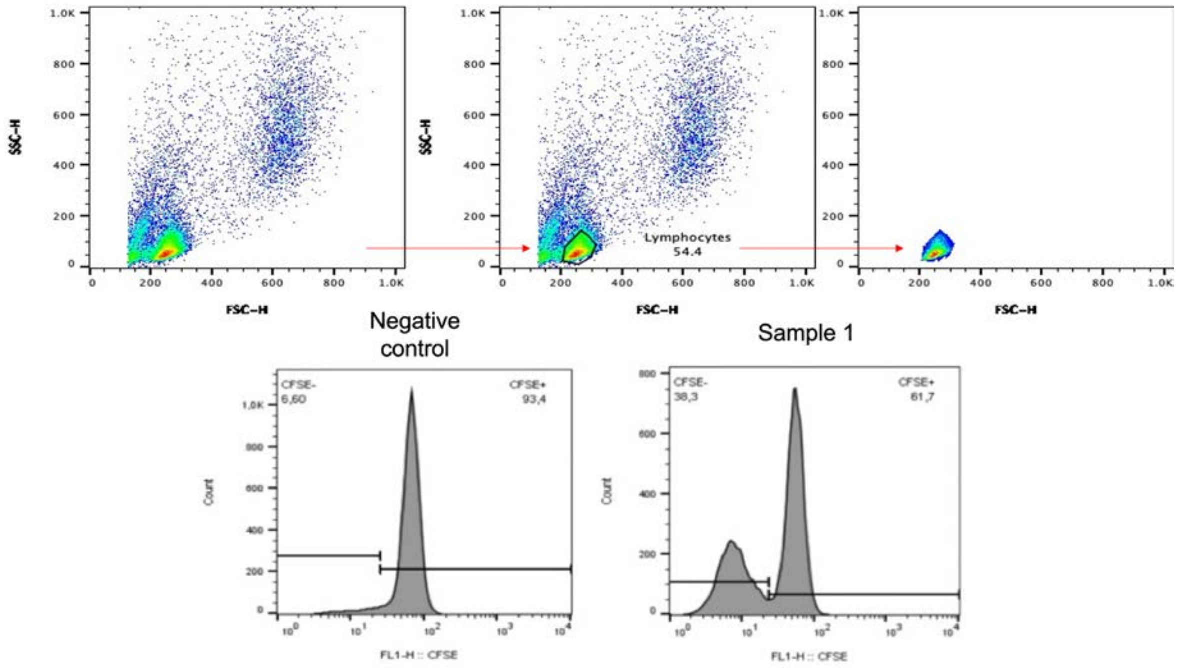


**Figure S2.** Gating strategy for proliferation assay using CFSE.

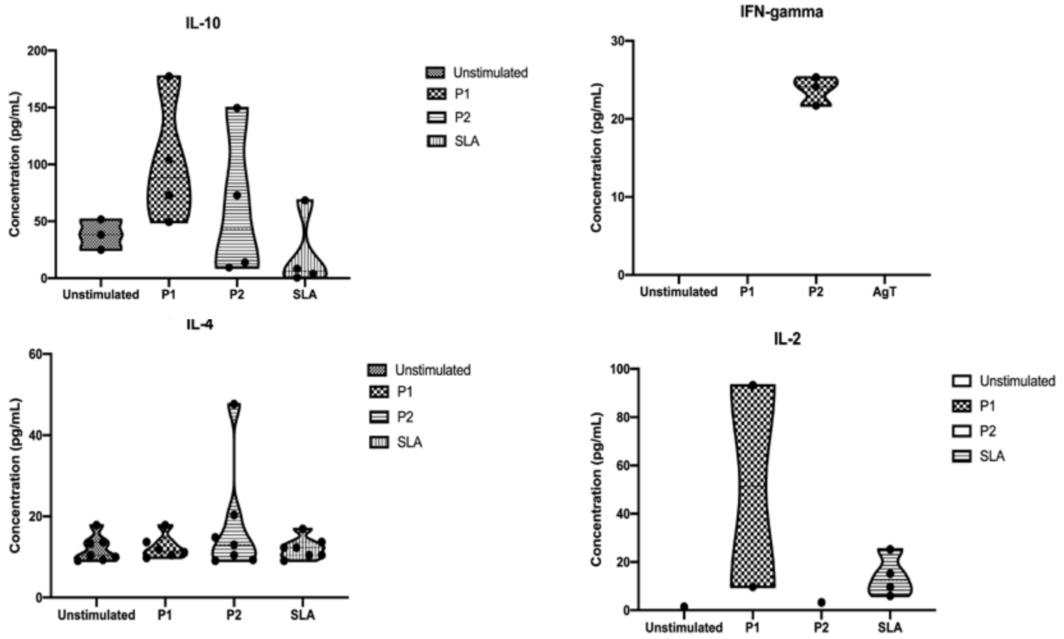


**Figure S3.** Th1 and Th2 cytokine production on CL group after 48h stimulation. P1– pool 1, P2 – pool 2, SLA – Soluble *Leishmania* antigen.

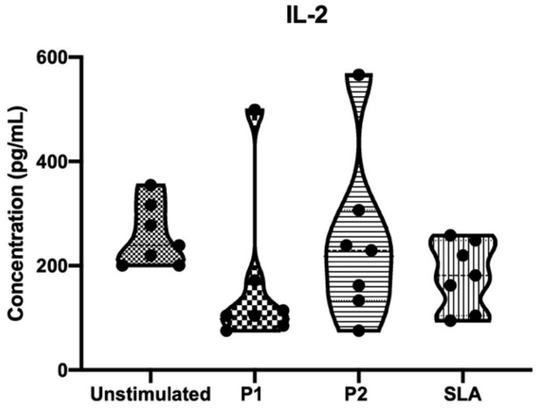


**Figure S4.** Th1 and Th2 cytokine production on CT group after 48h stimulation. P1– pool 1, P2 – pool 2, SLA – Soluble *Leishmania* antigen.
